# Social capital and self-rated health among adolescents in Brazil: an exploratory study

**DOI:** 10.1186/1756-0500-3-338

**Published:** 2010-12-16

**Authors:** Carolina M Borges, Ana Cristina V Campos, Andrea D Vargas, Efigênia F Ferreira, Ichiro Kawachi

**Affiliations:** 1 Department of Social and Preventive Dentistry, Federal University of Minas Gerais, 6627 Presidente Antônio Carlos Ave, Belo Horizonte, MG 31270-901, Brazil; 2 Department of Society, Human Development and Health, Harvard School of Public Health, 677 Huntington Ave, Boston MA 02115, USA

## Abstract

**Background:**

Social capital may influence health and the patterns of association differ according its dimension such as cognitive, behavioral, bridging or bonding. There is a few numbers of studies in Latin America which comprise these aspects of social capital and health. The aim of this study was to examine the association between social capital and self-rated health among youth, and distinguish between the different forms of social capital - cognitive versus behavioral, and bonding versus bridging.

**Findings:**

A cross-sectional study was conducted in 2009 among working adolescents supported by a Brazilian NGO. The sample comprised 363 individuals and data were collected using a validated structured questionnaire. The outcome, self-rated health, was measured as a dichotomous variable (poor/good health) and fourteen social capital indicators were investigated (cognitive, behavioral and bonding/bridging). Data were analyzed using multivariate logistic regression. Cognitive (social support and trust), behavioral (civic participation) and bridging social capital were associated with good self-rated health after adjustment of all the other social capital indicators and confounders (sex, age, skin color and educational background).

**Conclusions:**

Social capital was associated with self-rated health and the patterns of association differed according its specific dimensions. Cognitive, behavioral and bridging social capitals were protective for adolescents health living in a developing country context..

## Background

Social capital can be understood as features of social structures - including norms, inter-personal trust, and mutual support - which act as resources for individuals and also facilitate collective action [1,2]. Although the concept originated in sociology to explain diverse phenomena such as educational success, labor market attachment, and the prevention of crime, an increasing number of studies in the last decade and a half have turned to the exploration of social capital in public health [3]. To date, studies have suggested that social capital may be a determinant of health based on its association with mortality [4], health behaviors [5], mental health [6] and self-rated health [7,8].

Several mechanisms have been suggested by which social capital can influence health [9]. It is important to note that the influence of social capital on health can be either health-promoting or health-damaging, depending on the social context. Thus, social capital can enhance the diffusion of deleterious health practices such as the spread of smoking among adolescent social networks via social learning [10]. On the other hand in other situations, such as school-based, peer-led interventions, social capital can enhance the diffusion of health-promoting behaviors such as smoking cessation. A second mechanism by which social capital can influence health is through the provision and exchange of mutual psychosocial support. Again, the effects can be either health-promoting or health-damaging depending on the social context. Thus, it has been documented that involvement in dense social ties can be a detriment to health in resource-deprived settings stemming from the excess burden of providing social support to others [11]. By contrast, mutual support and norms of reciprocity have been found to be protective for mental and physical health in settings where basic individual needs are already met. Yet a third mechanism by which social capital can influence health is via the exercise of collective action and informal social control [12]. Groups that are rich in stocks of social capital have greater capacity (i.e. the "collective efficacy") to bring about desired outcomes such as the prevention of antisocial behaviors like drug abuse among minors in the community. On the other hand, a community that is too cohesive can lead to pressure to conform as well as ostracism of individuals who behave differently from the others [13]. The challenge in social capital research is thus to identify and isolate the specific instances in which it can be a force for health promotion versus a detrimental factor in the patterning of health outcomes.

By now an established convention in social capital research is to distinguish between the impacts of the so-called cognitive dimension of social capital - e.g. perceived trust of others - versus the behavioral dimension - such as participation in groups [14]. Increasingly the distinction is also drawn between "bonding" social capital and "bridging" social capital. Bonding capital refers to the resources that accrue to members of a group who are similar to each other with respect to social identity, such as class, race/ethnicity, or religion. By contrast, bridging capital refers to resources that span across social cleavages [15]. Although very few empirical studies in the health field have addressed this distinction, research suggests that bonding capital can be both promoting and damaging for health (depending on the context), whereas bridging capital seems to be more uniformly protective for health because of the potential to link resource-deprived individuals to material and symbolic resources.

In the present study, based on an adolescent sample in Brazil, we sought to address three questions. First, we sought to focus on the association of social capital with self-rated health among youth. The bulk of empirical studies to date have been conducted among adult samples, and less is known about the links between social capital and health outcomes among adolescents [16,17]. Secondly, we sought to distinguish between the different dimensions of social capital-cognitive versus behavioral, and bonding versus bridging. Lastly, we focused on the Latin American context where few studies have been conducted on social capital so far. In a systematic review of studies conducted throughout the world, Islam and colleagues [18] noted that the association between social capital and health tended to be stronger, and more consistently observed, in societies characterized by unequal distribution of social resources (e.g. the United States). By contrast, among more egalitarian societies (such as the Nordic welfare states), social capital was less consistently associated with health outcomes. Brazil is a very unequal society and we hypothesized that having access to social capital could account for variation in the health status of adolescents.

## Material and methods

We carried out a cross-sectional study in 2009 in a town that belongs to the metropolitan urban area of Belo Horizonte, capital of Minas Gerais state, Brazil.

Our sample of adolescents was drawn from a philanthropic non-governmental organization (NGO) which was established in 1966 with the purpose of connecting working adolescents to local employers. The adolescents self-refer to the services of the NGO, which maintains the following admission criteria: a) the adolescents must be aged between 16 years to 17 years and 11 months, b) they must be students enrolled in a public school, c) they must come from economically vulnerable family backgrounds, and d) they must take and complete the admission course provided by the NGO. Neither race nor religious backgrounds are taken into consideration at admission. During the admission course, the youth have an opportunity to learn about the NGO statute and its bylaws, to attend Portuguese and reading classes, and to be prepared to fit in the companies that will hire them.

By the end of the course, all the adolescents receive a qualification certificate on human relations, work team and professional behaviors. Once accepted by the NGO, the youth have an opportunity to get a part-time job and also to attend free sports classes and extra recreational activities on weekends.

The recruitment of the participants required one day and the adolescents recruited in this present study were all those present during a special weekend meeting and who had signed assent to participate in the survey (and from whom we also obtained consent from either their parents or guardians). For this present study, we used the World Bank Integrated Questionnaire to Measure Social Capital (SC-QI), which is a psychometrical validated instrument [19]. The World Bank group study distributed some indicators into dimensions based on their previous experience with social capital survey and reading scientific literature. This instrument has 27 items and is divided into six different dimensions of social capital as follow: a) groups and network, b) trust and solidarity, c) collective action and cooperation, d) information and communication, e) social cohesion and inclusion, f) empowerment and political action. The IQ-SC questionnaire was conceptualized for the micro level (individuals) and does not collect data on the level of communities, thus it is an adequate instrument for the objective of our study.

The IQ-SC has no overall scoring algorithm. This explains the possibility of our selection of some social capital indicators among the total 27. One of co-authors (I.K.) is an expert on social capital and health studies and selected 14 of 27 items based on social epidemiological evidence and the peculiarities of adolescence age group.

All the adolescents in the NGO (N = 363) participated in the survey. Ethics approval was granted by Research Ethics Committee at Pontificia Universidade Catolica de Minas Gerais.

### Outcome Measure

Our outcome measure was a one-item self-rated overall health status question. Individuals responded to the following question: "How would you describe your overall state of health these days, would you say it is very good, good, fair, poor or very poor?". We combined the categories to have a binary outcome of self-rated health where 0 = very good, good or fair and 1 = poor or very poor.

### Social Capital

The cognitive dimensions of social capital were assessed by four questions inquiring about: a) trust in others, b) perceived helpfulness of neighbors and c) perceptions of whether the youth could borrow money from others in case of need. The variable "trust in people" was dichotomized as: 0 = people can be trusted or 1 = you can't be too careful. The variable "helpfulness of neighbors" was assessed by the extent to which youth agreed with the 2 statements that: "most people in this neighborhood are willing to help you in case of need" and "in this neighborhood, one has to be alert or someone is likely to take advantage of you". The answers (agree strongly, agree somewhat, neither agree nor disagree, disagree somewhat, disagree strongly) were dichotomized into 0 = agree versus 1 = disagree or unsure. The variable "borrow money" was determined by a five scale question asking if there was someone beyond family or close relatives willing to help the youth in case of need to borrow a small amount of money (definitely, probably, unsure, probably not or definitely not), which was dichotomized to 0 = yes and 1 = no or unsure.

The behavioral dimensions of social capital were assessed by: a) participation in community activities during the past twelve months; b) time or money contribution to a community project (0 = yes versus 1 = no); c) whether they belonged to a group (0 = at least one or more, 1 = zero); d) whether they had a close friend, and e) whether they got together with people to have food or drink in the past month 0 = at least one or more, versus 1 = no.

Finally, we assessed bonding versus bridging social capital by asking respondents to think about the people with whom they associated. Separate questions inquired whether the youth associated with others from different ethnic backgrounds, different economic or social status or different religious groups. For each of these questions, the responses were coded as 0 = yes (bridging) and 1 = no (bonding).

### Confounders

The variables sex (male or female), age (0 for 17 years old vs. 1 for 15-17 years old), skin color (white, black, brown, yellow, Indian - classification of Brazilian Institute of Geographic and Statistic) and educational background (first or second grade of middle school - correspond to the 11^th ^grade American high school) were entered as possible confounders in regression models.

### Statistical Analyses

The SPSS statistical package version 18.0 was used for data analyses. Descriptive statistics were performed to characterize the participants including relative and absolute frequencies of the explanatory variables and confounders as well their associated odds ratio for poor self-rated health with 95% confidence intervals (95% CI).

Next, the Enter method was used for the logistic regression models. Variables with a *p*-value lower than 0.2 were included in the logistic regression models. Although social demographic variables did not meet the formal inclusion criterion [(sex p = 0.510), (age p = 0.714), (educational background p = 0.725), (skin color p = 0.515)], they were nonetheless retained in the regression models due to their theoretical plausibility.

## Results

Of the total respondents who took part in our study (N = 363), 95.9% were male and 4.1% were female. Regarding age, the proportion of 15-16 and 17 years old was similar, 53.3% and 46.7%, respectively. Fifty-seven percent of the adolescents reported their skin color as yellow (Asiatic) and 19.3% as black; 88.7% of the adolescents were enrolled in the second grade of Brazilian high school (Table 1 - Additional file 1).

Table 1 (Additional file 1) shows the descriptive statistics for individual variables and their crude odds ratios for poor self-rated health. Individuals who agreed that "one has to be alert or someone is likely to take advantage of he/she" were more likely to report poor self-rated health (OR = 2.2, 95% CI = 1.1-4.5) compared to those who did not express this sentiment. Individuals who did not get together to have food or drink with people of different social status (i.e. lacking bridging social capital) were more likely to report poor health (OR = 2.0, 95% CI = 1.1-3.5) compared to who did have a different opportunity. Contrary to expectation, however, adolescents who did not belong to a group had a 60% lower odds ratio (OR = 0.4, 95% CI = 0.2-0.9) of reporting poor self-rated health. The remaining indicators of social capital (e.g. trust, having someone to borrow money from, perception of reciprocity) were each associated with self-rated health in the expected direction (i.e. low perception of social capital = increased odds of poor health), but the associations were not statistically significant.

The first step of multivariable-adjusted logistic regression (*Model I*) was controlled for socioeconomic and demographic variables. Model II was controlled for Model I and all social capital indicators. The variable "got together to have drink/food" was constant for selected cases thus excluded from logistic regression. Among socio-demographic variables, Indian ethnic background was the only variable that was statistically significantly associated with an increased odds ratio of poor health. Four of the fourteen indicators of social capital were significantly associated with self-rated health. Adolescents who reported having no one to borrow money from (OR = 2.1, 95% CI = 1.1-4.3) and who agreed that someone was likely to take advantage of them (OR = 2.9, 95% CI = 1.2-7.2) were at increased risk of poor-self-rated health. Distrust (OR = 2.0, 95% CI = 0.2-19.2) and to believe that people are not willing to help in case of need (OR = 1.9, 95% CI = 0.9-3.9) increased the chance of reporting poor health although not statistically significant (Table 2 - Additional file 2).

The behavioral dimension of social capital was also associated with self-rated health - although in opposite directions depending on the indicator. Adolescents who did not contribute time to a community project had increased risk of poor self-rated health (OR = 1.9, 95% CI = 1.1-3.7). But by contrast, adolescents who reported not belonging to a group were at lower risk of poor self-rated health (OR = 0.5, 95% CI = 0.2-1.4). Being involved in communal activities was not associated with adolescents' health. Youth who did not contribute money to a community project (OR = 1.6, 95% CI = 0.8-3.1) and who did not have a close friend were more likely to report poor health when compared with those did (Table 2 - Additional file 2).

Of the three different types of bridging social capital that we inquired about (getting together with people of different economic status, social status, and race/ethnicity), only bridging relationships with people of different social status was significantly associated with self-rated health. Adolescents who did not get together with people of different social status were at 2.3 times the risk of poor self-rated health (95% CI: 1.1-5.2) compared with those who did. Lastly, Model III was adjusted for Model I and the four social capital indicators that were remained associated with the outcome (borrow money, advantage, time contribution to a community project and get together with people of different social status).

## Discussion

The objective of this study was to analyze a possible association between self-rated overall health and social capital among adolescents using four aspects of social capital (cognitive, behavioral and bonding/bridging). Our findings showed that social support, trust, civic participation and bridging social capital still remained associated with self-rated health after adjustment of all the other social capital indicators and confounders.

Adolescents who said that they had no one to borrow a small amount of money, who believed that someone else in the neighborhood would be likely to take advantage of them, who did not contribute time to a community project, and who did not get together with people from different social status had a greater likelihood of reporting poor or very poor health.

These findings converge with a previous study conducted in a developing country context. Khawaja and colleagues (2006) [17] investigated social capital and health status among 13-19 years old adolescents in Beirut addressing a similar question as our current study. Their results showed that distrust and social fragmentation were prevalent among individuals living in impoverished suburban communities; additionally, adolescents with low levels of social capital were almost four times more likely to report poor health compared to the others. Similar results were found in affluent countries such as Canada where youths from families of low affluence and low levels of social capital were more likely to report poor health [16].

In our study, the majority of respondents (95.8%) agreed that someone would likely to take advantage of them and almost 36% reported that there was no one beyond family or close relatives willing to help them in case of need to borrow a small amount of money. Capacity to trust could be seen as a consequence of personal trust which is related to connectedness in family and community as well [20]. Distrust and absent of reciprocity were associated with poor self-rated health among residents of a low income community nested in a Latin America country [11]. Our results were somewhat expected if we take into to account the reality of the actual structure of Brazilian society characterized by its inherent socioeconomic inequalities with huge gaps among different social classes and also a general distrust both in people and in government.

Bridging relations with people with different social status showed a positive effect on self-rated health, which may be a marker of the ability of adolescents to access valued resources held by individuals from higher social strata. While bonding social capital may be essential for emotional and social support as a whole, it does not enable the redistribution of resources from the advantaged to the disadvantaged groups [21]. Thus bridging social capital is important in enabling people from disadvantage groups to access materials and psychosocial resources and there is growing evidence that bridging social capital is positively associated with self-rated health, especially among disadvantaged groups [22,23].

With regard to civic participation, in Brazil there are a limited number of youth-oriented civic engagement and extracurricular activities through school and community-based youth organizations. Duke and colleagues (2009) [24] found that adolescents with strong familial relationships and community connections may experience healthy development and promote future civic engagement. Thus, connections at a young age contribute to important elements of social capital.

Some efforts have been done to try to increase stocks of social capital among adolescents in developing countries through controlled trials of interventions but these efforts are still in their infancy [25]. Empowerment, which is considered one of social capital dimensions, was studied by Pattussi and colleagues [26] among Brazilian adolescents. The multilevel analyses revealed that neighborhood empowerment may explain inequalities in the levels of dental caries. Adolescents from areas with higher levels of empowerment scores had lower levels of dental caries when compared with the adolescents whom lived in areas with low level of empowerment [26] and especially among boys [27].

### Study limitations

The cross-sectional nature of the data does not allow any conclusion concerning causal intercourse between overall self-rated health and social capital. It is important to highlight the possibility of reverse causation and reporting bias once social capital may influence health and vice-versa. For instance it has been pointed out that the lower stock of social capital lead to poor levels of health of populations. However, the opposite may occur since individuals with poor health could generate lower levels of social capital. Another limitation of this study is the inherent bias in self-reporting both self-rated health and social capital (common method variance). Additionally, given that social capital is often conceptualized as a group resource, the ideal study design would be multi-level, i.e. individuals nested within different contexts (e.g. neighborhoods) with different levels of social capital [28]. Unfortunately, we did not collect information on the areas in which the adolescents resided.

Our sample was limited in terms of socioeconomic variability, thus reducing the generalizability of our findings. The main pre-requisites for the adolescents' application to the NGO are that they must be from disadvantaged socioeconomic backgrounds and they must be enrolled at high school. These two requirements explain the comparative homogeneity of our sample. The majority of adolescents were males due the type of jobs offered by the employers, i.e. issuing parking tickets on streets.

Lastly, it is important to highlight that only four out of fourteen social capital items are significantly associated to self-rated health. This fact could indicate the absence of a robust social capital effect rather than particular social capital specificities or might indicate a power problem of our sample size.

### Study strengths

One of the strengths of our study is that we assessed all three dimensions of social capital - including the cognitive, behavioral and bonding/bridging aspects. Our findings underscore the point that specific indicators of social capital may have different associations with health outcomes.

Our study is also among the few so far that has been carried out in a developing country marked by wide socioeconomic disparities.

## Conclusion

Social capital may play an important role explaining better self-rated health and the pattern of association differs according its specific dimensions. The three specific dimensions - cognitive, behavioral and bridging social capital - seem to be protective for the health of working adolescents living in a developing country context such as Brazil.

## Competing interests

The authors declare that they have no competing interests.

## Authors' contributions

IK idealized the design of this study, drafted all its versions and did the critical and final review. CB idealized the design of the study, did statistical analysis and wrote the manuscript. AC collected the data and with EF and AV gave important contributions during all stages of the draft. All authors read and approved the final draft.

## Supplementary Material

Additional file 1 **"Table 1. Characteristics of respondents and their associated odds ratio for poor self-rated health (n = 363)"**. Table presenting the first part of results. Click here for file

Additional file 2 **"Table 2. Association of socio-demographic variables and social capital indicators with poor (regular/poor/very poor) self-rated health determined by logistic regression (n = 294)"**. Table presenting the second part of results. Click here for file
