# Supplementary material for: Social capital and self-rated health among adolescents in Brazil: an exploratory study
Source: BMC Res Notes. 2010 Dec 16;3:338. doi: 10.1186/1756-0500-3-338 (PMC3014966; doi:10.1186/1756-0500-3-338)
Supplement: Additional file 1 — "Table 1. Characteristics of respondents and their associated odds ratio for poor self-rated health (n = 363)". Table presenting the first part of results. [file 1756-0500-3-338-S1.DOCX]

| Table 1. Characteristics of respondents and their associated odds ratio for poor self-rated health (n=363). | | | | | | |
| --- | --- | --- | --- | --- | --- | --- |
|  |  |  | **Poor self-rated overall health** | | | |
| **Variable** | **Category** |  | **N (%)** | **N cases (%)** | **Crude odds ratio** | **95%CI** |
| Sex (n=362) | Male |  | 347 (95.9) | 68 (19.5) | 1.0 (ref) |  |
|  | Female |  | 15 (4.1) | 04 (26.6) | 1.4 | [0.4-4.8] |
| Age (n=362) |  |  |  |  |  |  |
|  | 17 years-old |  | 169 (46.7) | 35 (20.7) | 1.0 (ref) |  |
| . | 15-16 years-old |  | 193 (53.3) | 37 (19.1) | 0.9 | [05-1.5] |
| Skin color (n=360) |  |  |  |  |  |  |
|  | White |  | 50 (13.8) | 09 (18.0) | 1.0 (ref) |  |
|  | Black |  | 67 (19.3) | 13 (19.4) | 1.1 | [0.4-2.8] |
|  | Brown |  | 20 (5.5) | 04 (20.0) | 1.1 | [0.3-4.2] |
|  | Yellow |  | 207 (57.0) | 40 (19.3) | 1.1 | [0.4-2.4] |
|  | Indian |  | 16 (4.4) | 06 (37.5) | 2.7 | [0.7-9.4] |
| Educational background (n=362) |  |  |  |  |  |  |
|  | First grade (HS) |  | 41 (11.3) | 09 (21.9) | 1.0 (ref) |  |
|  | Second grade (HS) |  | 321 (88.7) | 63 (19.6) | 0.8 | [0.3-1.9] |
| Borrow Money (n=360) |  |  |  |  |  |  |
|  | Yes |  | 231 (64.2) | 37 (16.0) | 1.0 (ref) |  |
|  | No; Unsure |  | 129 (35.8) | 33 (25.6) | 1.8 | [1.0-3.0] |
| Trust (n=358) |  |  |  |  |  |  |
|  | Yes |  | 15 (4.2) | 02 (13.3) | 1.0 (ref) |  |
|  | Can’t be too carefull |  | 343 (95.8) | 70 (20.4) | 1.6 | [0.4-7.5] |
| People are willing to help you (n=361) |  |  |  |  |  |  |
|  | Yes |  | 160 (44.3) | 26 (16.3) | 1.0 (ref) |  |
|  | No; Unsure |  | 201 (55.7) | 45 (22.4) | 1.4 | [0.8-2.5] |
| Advantage (n=360) |  |  |  |  |  |  |
|  | Disagree |  | 96 (26.6) | 11 (11.5) | 1.0 (ref) |  |
|  | Agree somewhat/strongly |  | 264 (73.4) | 60 (22.2) | 2.2 | [1.1-4.5] |
| Communal activities past 12 months (n=358) |  |  |  |  |  |  |
|  | Yes |  | 108 (30.2) | 22 (20.4) | 1.0 (ref) |  |
|  | No |  | 250 (69.8) | 50 (20.0) | 0.9 | [0.5-1.7] |
| Money contribution to community project (n=347) |  |  |  |  |  |  |
|  | Yes |  | 202 (58.2) | 36 (17.8) | 1.0 (ref) |  |
|  | No |  | 145 (41.8) | 33 (22.8) | 1.3 | [0.8-2.3] |
| Time contribution to community project (n=353) |  |  |  |  |  |  |
|  | Yes |  | 177 (50.1) | 27 (15.3) | 1.0 (ref) |  |
|  | No |  | 176 (49.9) | 41 (23.3) | 1.6 | [0.9-2.8] |
| Belong to a group (n=359) |  |  |  |  |  |  |
|  | Yes (≥ 01 group) |  | 284 (79.1) | 63 (22.2) | 1.0 (ref) |  |
|  | No |  | 77 (20.9) | 09 (11.7) | 0.4 | [0.2-0.9] |
| Have a close friend (n=359) |  |  |  |  |  |  |
|  | Yes (≥ 01) |  | 355 (98.8) | 71 (20.0) | 1.0 (ref) |  |
|  | No |  | 04 (0.4) | 01 (25) | 1.3 | [0.1-13.0] |
| Got together to have drink/food (n=336) |  |  |  |  |  |  |
|  | Yes (≥ 01 time) |  | 311 (92.5) | 60 (19.3) | 1.0 (ref) |  |
|  | No |  | 25 (07.5) | 09 (36.0) | 2.3 | [0.9-5.5] |
| Got together people different race (n=329) |  |  |  |  |  |  |
|  | Yes |  | 189 (57.4) | 37 (19.6) | 1.0 (ref) |  |
|  | No |  | 140 (52.6) | 26 (18.6) | 1.1 | [0.6-1.8] |
| Got together people different economic status (n=327) |  |  |  |  |  |  |
|  | Yes |  | 210 (64.2) | 43 (20.5) | 1.0 (ref) |  |
|  | No |  | 117 (35.8) | 20 (17.1) | 1.2 | [0.6-2.2] |
| Got together people different social status (n=327) |  |  |  |  |  |  |
|  | Yes |  | 157 (48.0) | 39 (24.8) | 1.0 (ref) |  |
|  | No |  | 170 (52) | 24 (14.1) | 2.0 | [1.1-3.5] |
| Got together people different religion (n=326) |  |  |  |  |  |  |
|  | Yes |  | 193 (59.2) | 40 (20.7) | 1.0 (ref) |  |
|  | No |  | 133 (40.8) | 23 (17.3) | 1.2 | [0.7-2.2] |
