# Supplementary material for: Social capital and self-rated health among adolescents in Brazil: an exploratory study
Source: BMC Res Notes. 2010 Dec 16;3:338. doi: 10.1186/1756-0500-3-338 (PMC3014966; doi:10.1186/1756-0500-3-338)
Supplement: Additional file 2 — "Table 2. Association of socio-demographic variables and social capital indicators with poor (regular/poor/very poor) self-rated health determined by logistic regression (n = 294)". Table presenting the second part of results. [file 1756-0500-3-338-S2.DOCX]

Table 2. Association of socio-demographic variables and social capital indicators with poor (regular/poor/very poor) self-rated health determined by logistic regression (n=294).

| **Variable** | ***Model I ^a^*** | | |  | ***Model II ^b^*** |  | ***Model III ^c^*** | | |
| --- | --- | --- | --- | --- | --- | --- | --- | --- | --- |
|  | OR* | [CI 95%] | P | OR* | [CI 95%] | P | OR* | [CI 95%] | P |
| **Sex** |  |  | 0.540 |  |  | 0.120 |  |  | 0.235 |
| Male | 1.0 |  |  | 1.0 |  |  | 1.0 |  |  |
| Female | 1.4 | 0.4-4.7 |  | 3.0 | 0.7-12.7 |  | 2.2 | 0.5-8.5 |  |
| **Age** |  |  | 0.648 |  |  | 0.895 |  |  | 0.997 |
| 17 | 1.0 |  |  | 1.0 |  |  | 1.0 |  |  |
| 15-16 | 0.8 | 0.5-1.4 |  | 1.1 | 0.5-2.0 |  | 0.9 | 0.5-1.8 |  |
| **Skin color** |  |  | 0.548 |  |  | 0.157 |  |  | 0.128 |
| White | 1.0 |  |  | 1.0 |  |  | 1.0 |  |  |
| Black | 1.1 | 0.4-2.8 |  | 2.2 | 0.6-7.9 |  | 1.8 | 0.6-5.8 |  |
| Brown | 1.1 | 0.2-4.1 |  | 2.5 | 0.5-12.6 |  | 2.0 | 0.4-9.5 |  |
| Yellow | 1.1 | 0.4-2.4 |  | 1.5 | 0.5-4.6 |  | 1.2 | 0.4-3.4 |  |
| Indian | 2.7 | 0.7-9.4 |  | 7.2 | 1.3-38.3 |  | 6.1 | 1.3-28.1 |  |
| **Educational Background** |  |  | 0.767 |  |  | 0.459 |  |  | 0.785 |
| 1st grade High School | 1.0 |  |  | 1.0 |  |  | 1.0 |  |  |
| 2d grade High School | 1.1 | 0.5-2.5 |  | 0.6 | 0.2-1.9 |  | 0.8 | 0.3-2.3 |  |
| **Borrow money** |  |  |  |  |  | 0.027 |  |  | 0.010 |
| Yes |  |  |  | 1.0 |  |  | 1.0 |  |  |
| No |  |  |  | 2.1 | 1.1-4.3 |  | 2.3 | 1.2-4.4 |  |
| **Someone is likely to take advantage of you** |  |  |  |  |  | 0.017 |  |  | 0.010 |
| Disagree |  |  |  | 1.0 |  |  | 1.0 |  |  |
| Agree |  |  |  | 2.9 | 1.2-7.2 |  | 2.9 | 1.2-6.9 |  |
| **Time contribution to community project** |  |  |  |  |  | 0.054 |  |  | 0.027 |
| Yes |  |  |  | 1.0 |  |  | 1.0 |  |  |
| No |  |  |  | 1.9 | 1.1-3.7 |  | 2.1 | 1.1-3.9 |  |
| **Got together people different social status** |  |  |  |  |  | 0.030 |  |  | 0.003 |
| Yes |  |  |  | 1.0 |  |  | 1.0 |  |  |
| No |  |  |  | 2.3 | 1.1-5.2 |  | 2.6 | 1.4-5.1 |  |
| **Belong to a group** |  |  |  |  |  | 0.247 |  |  |  |
| Yes (at least one) |  |  |  | 1.0 |  |  |  |  |  |
| No |  |  |  | 0.5 | 0.2-1.4 |  |  |  |  |
| **Trust**  Yes  You can’t be too careful |  |  |  | 1.0  2.0 | 0.2-19.2 | 0.526 |  |  |  |
| **People are willing to help you** |  |  |  |  |  | 0.061 |  |  |  |
| Yes |  |  |  | 1.0 |  |  |  |  |  |
| No |  |  |  | 1.9 | 0.9-3.9 |  |  |  |  |
| **Communal activities past 12 months** |  |  |  |  |  | 0.976 |  |  |  |
| Yes |  |  |  | 1.0 |  |  |  |  |  |
| No |  |  |  | 1.0 | 0.5-2.0 |  |  |  |  |
| **Money contribution to community project** |  |  |  |  |  | 0.148 |  |  |  |
| Yes |  |  |  | 1.0 |  |  |  |  |  |
| No |  |  |  | 1.6 | 0.8-3.1 |  |  |  |  |
| **Have a close friend** |  |  |  |  |  | 0.804 |  |  |  |
| Yes (≥ 01) |  |  |  | 1.0 |  |  |  |  |  |
| No |  |  |  | 1.3 | 0.1-13.0 |  |  |  |  |
| **Got together people different race** |  |  |  |  |  | 0.680 |  |  |  |
| Yes |  |  |  | 1.0 |  |  |  |  |  |
| No |  |  |  | 0.8 | 0.4-1.7 |  |  |  |  |
| **Got together people different economic status** |  |  |  |  |  | 0.635 |  |  |  |
| Yes |  |  |  | 1.0 |  |  |  |  |  |
| No |  |  |  | 0.8 | 0.3-1.8 |  |  |  |  |
| **Got together people different religion** |  |  |  |  |  | 0.630 |  |  |  |
| Yes |  |  |  | 1.0 |  |  |  |  |  |
| No |  |  |  | 1.1 | 0.5-2.4 |  |  |  |  |

^a^ Adjusted for socioeconomics characteristics (age, sex, self-reported skin color and educational background)

^b^ Adjusted for Model I and social capital variables; the variable “got together to have drink/food” was excluded from regression was constant for selected cases.

^c^ Social capital indicators statistically significant and confounders.

* Adjusted odds ratio

Considering prevalence of poor self-rated health = 20%
